# Early dynamic immune-inflammatory and nutritional recovery signature is associated with durable benefit and early progression in advanced non-small cell lung cancer receiving first-line PD-1/PD-L1 inhibitor-based therapy: a real-world retrospective cohort study

**DOI:** 10.3389/fmed.2026.1900721

**Published:** 2026-08-14

**Authors:** Tingting Zhan, Hang Cao

**Affiliations:** Department of Oncology, The Third People’s Hospital of Chengdu, Chengdu, China

**Keywords:** advanced lung cancer inflammation index, durable clinical benefit, early progression, immune checkpoint inhibitor, neutrophil-to-lymphocyte ratio, non-small cell lung cancer, PD-1, PD-L1

## Abstract

**Background:**

Durable benefit from first-line PD-1/PD-L1 inhibitor-based therapy remains heterogeneous in advanced non-small cell lung cancer (NSCLC). Baseline biomarkers, including PD-L1 tumor proportion score, tumor mutational burden, and static inflammatory indices, only partly capture this heterogeneity and may not reflect host response. We evaluated whether a cycle 2 immune-inflammatory and nutritional recovery signature from blood markers was associated with durable clinical benefit (DCB), early progression, and survival.

**Methods:**

This single-center retrospective cohort included 420 patients with advanced NSCLC treated with first-line PD-1/PD-L1 inhibitor-based therapy at Chengdu Third People’s Hospital between January 2019 and September 2025, with data cutoff on March 31, 2026. The primary analytic cohort comprised 409 patients with a rule-derived cycle 2 dynamic recovery signature: favorable recovery, intermediate, or persistent failure. DCB at 6 months and early progression within 3 months were the primary endpoints. Associations were estimated using Firth-type penalized logistic regression and Cox models adjusted for clinical covariates and baseline NLR, PNI, and ALI. Model performance was assessed using discrimination, calibration, decision curves, and bootstrap optimism correction.

**Results:**

Among 409 classifiable patients, 141 (34.5%), 115 (28.1%), and 153 (37.4%) had favorable recovery, intermediate, and persistent failure signatures. DCB occurred in 61.7, 43.5, and 33.3%, and early progression occurred in 11.3, 18.3, and 23.5%, respectively. Compared with favorable recovery, persistent failure was associated with lower DCB (adjusted odds ratio [OR], 0.34; 95% CI, 0.21–0.56), higher early progression (adjusted OR, 2.15; 95% CI, 1.13–4.07), and shorter progression-free survival (adjusted hazard ratio [HR], 3.34; 95% CI, 2.40–4.64). Its adjusted association with overall survival was weaker (HR, 1.22; 95% CI, 0.86–1.72). The clinical plus dynamic signature model had optimism-corrected AUCs of 0.619 for DCB and 0.562 for early progression. The composite signature did not outperform individual continuous dynamic biomarkers.

**Conclusion:**

A rule-derived cycle 2 immune-inflammatory and nutritional recovery signature was independently associated with DCB and progression-free survival, but discrimination was modest, early-progression performance was weak, and no robust adjusted overall survival association was observed. It should be interpreted as an exploratory risk-stratification summary, not a clinically ready prediction tool. External validation and prospective evaluation are required before clinical use.

## 1 Introduction

Lung cancer remains the leading cause of cancer mortality worldwide, and NSCLC accounts for most cases (1, 2). For patients with advanced disease without an actionable driver alteration, PD-1/PD-L1 inhibitor-based therapy has become a central first-line treatment strategy (3, 4). Randomized trials have established pembrolizumab monotherapy for tumors with high PD-L1 expression and pembrolizumab plus platinum-based chemotherapy across nonsquamous and squamous histologies (5–8). Long-term updates from KEYNOTE-189 and KEYNOTE-407 confirmed durable survival gains, but they also showed that a substantial proportion of patients progress early despite first-line chemoimmunotherapy (9, 10).

Existing predictive biomarkers only partly explain this heterogeneity. PD-L1 tumor proportion score is clinically useful, but responses occur in some PD-L1-low tumors and primary resistance occurs even when PD-L1 expression is high (5, 8). Tumor mutational burden provides an important biological rationale for immunotherapy sensitivity, yet its clinical use is constrained by assay variability, panel size, tissue availability, and inconsistent thresholds (11–14). Static peripheral blood indices share a similar limitation: they summarize pretreatment host status, but they do not capture whether the inflammatory and nutritional milieu changes after treatment has begun.

Peripheral blood markers are attractive because they are inexpensive, repeatable, and routinely available before each treatment cycle. A high NLR has been associated with poor outcomes in nivolumab-treated NSCLC and in meta-analyses of lung cancer patients receiving immune checkpoint inhibitors (15, 16). Biologically, NLR reflects the balance between neutrophil-associated inflammatory and immunosuppressive pressure and lymphocyte-mediated antitumor immune capacity; neutrophils can support tumor progression through cytokine signaling, angiogenesis, and suppression of cytotoxic T-cell activity, whereas lymphocyte infiltration is generally associated with favorable antitumor immunity (17, 18). Composite indices that combine systemic inflammation, LDH, albumin, body mass, or lymphocyte reserve have also been linked to immunotherapy outcomes, including LIPI, PNI, ALI, and CRP-based measures (19–23). Albumin is a marker of nutritional reserve and systemic inflammatory burden, and low serum albumin has been consistently associated with poor cancer outcomes (24).

Early on-treatment changes may be more informative than a single pretreatment value because they reflect the interaction between tumor control, treatment toxicity, systemic inflammation, and nutritional reserve. Dynamic changes in serum tumor markers and NLR have been reported to predict response to PD-1/PD-L1 inhibitors in advanced NSCLC (25). CRP kinetics have also been associated with response patterns during checkpoint inhibitor therapy, supporting the concept that early inflammatory adaptation can carry prognostic information (26). However, prior dynamic biomarker studies have generally evaluated single markers such as NLR or CRP separately. It remains uncertain whether a simple tiered signature that integrates both inflammatory and nutritional recovery, using prespecified rules and routine cycle 2 blood tests, can summarize risk for durable clinical benefit, early progression, and longer-term outcomes during first-line PD-1/PD-L1 inhibitor-based therapy.

We therefore conducted a real-world retrospective cohort study of advanced NSCLC patients treated with first-line PD-1/PD-L1 inhibitor-based therapy. The study evaluated a cycle 2 dynamic immune-inflammatory and nutritional recovery signature derived from NLR, PNI, and ALI changes, with DCB at 6 months and early progression within 3 months as primary endpoints. We also examined PFS, OS, safety outcomes, individual dynamic components, threshold and confounding sensitivity analyses, treatment-heterogeneity analyses, and internally validated model performance. The intended role of the signature was an interpretable exploratory risk-stratification summary based on tests already embedded in routine care, not a stand-alone tool for individual treatment decisions.

## 2 Methods

### 2.1 Study design and patients

This was a single-center retrospective cohort study conducted at the Department of Oncology, Chengdu Third People’s Hospital. The source dataset included 420 real-world patients with advanced NSCLC who received first-line PD-1/PD-L1 inhibitor-based systemic therapy between January 2019 and September 2025. The data cutoff date was March 31, 2026. Reporting followed the principles of the STROBE statement for observational cohort studies (27).

Patients were included in the analytic dataset if they had advanced unresectable stage IIIB/IIIC, recurrent, or stage IV NSCLC and received a first-line regimen containing a PD-1 or PD-L1 inhibitor. Patients with an actionable driver alteration were not excluded if they had received first-line PD-1/PD-L1 inhibitor-based therapy in routine practice; this subgroup was uncommon and was evaluated in sensitivity analysis. Patients without sufficient cycle 2 information to classify the dynamic recovery signature were retained in the cohort flow and missingness summaries but excluded from the primary adjusted comparisons of the three signature groups. No patient had a baseline albumin value lower than 20 g/L in the available dataset. Standardized fields for pre-existing autoimmune disease, severe baseline infection, antibiotic exposure, fever, procalcitonin, or baseline obstructive pneumonia were not consistently available, and these factors were therefore not used as exclusion criteria or primary covariates.

Treatment patterns included ICI plus chemotherapy, ICI monotherapy, ICI plus chemotherapy plus antiangiogenic therapy, or ICI plus antiangiogenic therapy. Treatment selection reflected routine clinical practice and was not protocolized. Because treatment choice may be related to PD-L1 TPS, performance status, tumor burden, histology, and prognosis, first-line treatment pattern was included as an adjustment variable in the primary multivariable models. Additional analyses evaluated ICI plus chemotherapy only, absence or presence of antiangiogenic therapy, and formal interaction terms for treatment pattern and antiangiogenic exposure.

### 2.2 Dynamic recovery signature

The main exposure was a rule-derived cycle 2 dynamic immune-inflammatory and nutritional recovery signature. The signature was based on three routine blood-derived changes from baseline to cycle 2: NLR percent change, PNI absolute change, and ALI percent change. NLR percent change was calculated as (cycle 2 NLR - baseline NLR)/baseline NLR x 100. PNI absolute change was calculated as cycle 2 PNI - baseline PNI. ALI percent change was calculated as (cycle 2 ALI - baseline ALI) / baseline ALI x 100.

Each of the three domains was classified as improved at cycle 2 if it met a prespecified minimal-change threshold relative to baseline: an NLR decrease of at least 15%, a PNI increase of at least 0.5 points, or an ALI increase of at least 15%. Favorable recovery denoted concordant early improvement, with all three domains meeting the improvement threshold. Persistent failure denoted absent early recovery, with none of the three domains meeting the improvement threshold. Intermediate denoted partial or mixed recovery, with one or two of the three domains meeting the improvement threshold. Patients with missing cycle 2 information required for signature assignment were labeled not classifiable.

The 15% relative thresholds for NLR and ALI and the 0.5-point absolute threshold for PNI were selected as prespecified, clinically interpretable minimal-change rules around a zero-change boundary rather than externally validated clinical cutoffs. Equal weighting was used to avoid outcome-derived weights and to preserve bedside interpretability. No threshold, component weight, or category assignment was selected by maximizing DCB, early progression, PFS, or OS, and no outcome-derived cutpoint search was performed. Threshold sensitivity analyses evaluated alternative rules of 10%/0/10, 20%/1/20, 15%/0/15, and 20%/0.5/20% for NLR decrease, PNI increase, and ALI increase, respectively.

### 2.3 Outcomes

The primary endpoints were DCB at 6 months and early progression within 3 months. DCB was defined as complete response, partial response, or stable disease maintained for at least 6 months without earlier progression, a disease-control endpoint used in immune checkpoint inhibitor studies and assessed according to RECIST version 1.1 (11, 28). DCB was selected because durable disease control is clinically meaningful in immunotherapy-treated patients; however, stable disease is biologically heterogeneous and was therefore interpreted as a clinical endpoint rather than a direct measure of tumor immune sensitivity. Early progression was defined as progression recorded within the first 3 months after treatment initiation, corresponding to the early radiographic reassessment window in routine practice and intended to capture primary resistance or rapidly progressive disease patterns described during PD-1/PD-L1 inhibitor therapy (29). Tumor response was assessed according to RECIST version 1.1, and best response categories were complete response, partial response, stable disease, progressive disease, or not evaluable (28). Objective response was defined as complete or partial response, and disease control was defined as complete response, partial response, or stable disease.

Secondary endpoints included PFS, OS, best RECIST response, objective response, disease control, any immune-related adverse event (irAE), immune-related pneumonitis, grade 3 or higher adverse events, and treatment discontinuation. PFS was calculated from treatment initiation to progression or death, and OS was calculated from treatment initiation to death from any cause. Patients without an event were censored at the last available follow-up.

### 2.4 Statistical analysis

Continuous variables are summarized as median and interquartile range, and categorical variables are summarized as number and percentage. Baseline characteristics were compared across signature groups using Kruskal-Wallis tests for continuous variables and chi-square or Fisher exact tests for categorical variables, as appropriate. Table *p* values indicate global between-group comparisons across the three classifiable signature categories. The primary adjusted analyses excluded patients with a not classifiable signature.

Binary endpoints were modeled using Firth-type penalized logistic regression to reduce instability from sparse clinical categories. Survival endpoints were analyzed using Cox proportional hazards models, and proportional hazards assumptions were evaluated with Schoenfeld residual tests. The primary multivariable models adjusted for age, sex, ECOG performance status, histology, stage, number of metastatic sites, brain metastasis, liver metastasis, PD-L1 TPS category, first-line treatment pattern, and baseline NLR, PNI, and ALI. Baseline NLR, PNI, and ALI were standardized before modeling. Patients with unknown PD-L1 TPS were retained as a separate category.

The dynamic signature was analyzed both as a categorical variable with favorable recovery as the reference group and as an ordinal variable to evaluate trend across favorable recovery, intermediate, and persistent failure. Individual dynamic components were analyzed per standard deviation increase. Sensitivity analyses repeated the persistent failure versus favorable recovery comparison in stage IV patients only, after excluding ICI monotherapy, and using a 1.5-month landmark approach for survival endpoints. Additional sensitivity analyses adjusted for hypertension and COPD, excluded patients with actionable driver alterations, excluded patients with unknown PD-L1 TPS, adjusted for baseline CRP in complete cases, evaluated the ICI plus chemotherapy subgroup, and evaluated patients without antiangiogenic therapy. Effect-modification tests were performed by adding interaction terms between the dynamic signature and PD-L1 TPS category, first-line treatment pattern, or antiangiogenic exposure.

Prediction analyses compared three prespecified models for DCB and early progression: a clinical model, a clinical plus baseline inflammatory/nutritional indices model, and a clinical plus dynamic signature model. Discrimination was assessed by ROC AUC with 95% confidence intervals. Calibration was evaluated by calibration slope and observed-versus-predicted plots by decile of predicted probability. Clinical utility was explored using decision curve analysis (30). Internal validation used 200 bootstrap resamples to estimate optimism-corrected AUC, calibration slope, and Brier score, consistent with prediction-model reporting principles (31, 32). Head-to-head prediction analyses compared the categorical composite signature with individual continuous dynamic NLR, PNI, and ALI changes and with a model containing all three continuous dynamic components. All analyses were performed in R version 4.5.3.

### 2.5 Ethics

The study was approved by the ethics committee of Chengdu Third People’s Hospital (approval No. cdth0125005). The dataset was derived from the hospital of the corresponding author and was analyzed retrospectively using de-identified clinical data. Because the study was retrospective and used only de-identified clinical data, the ethics committee waived the requirement for individual informed consent.

## 3 Results

**Figure 1:**
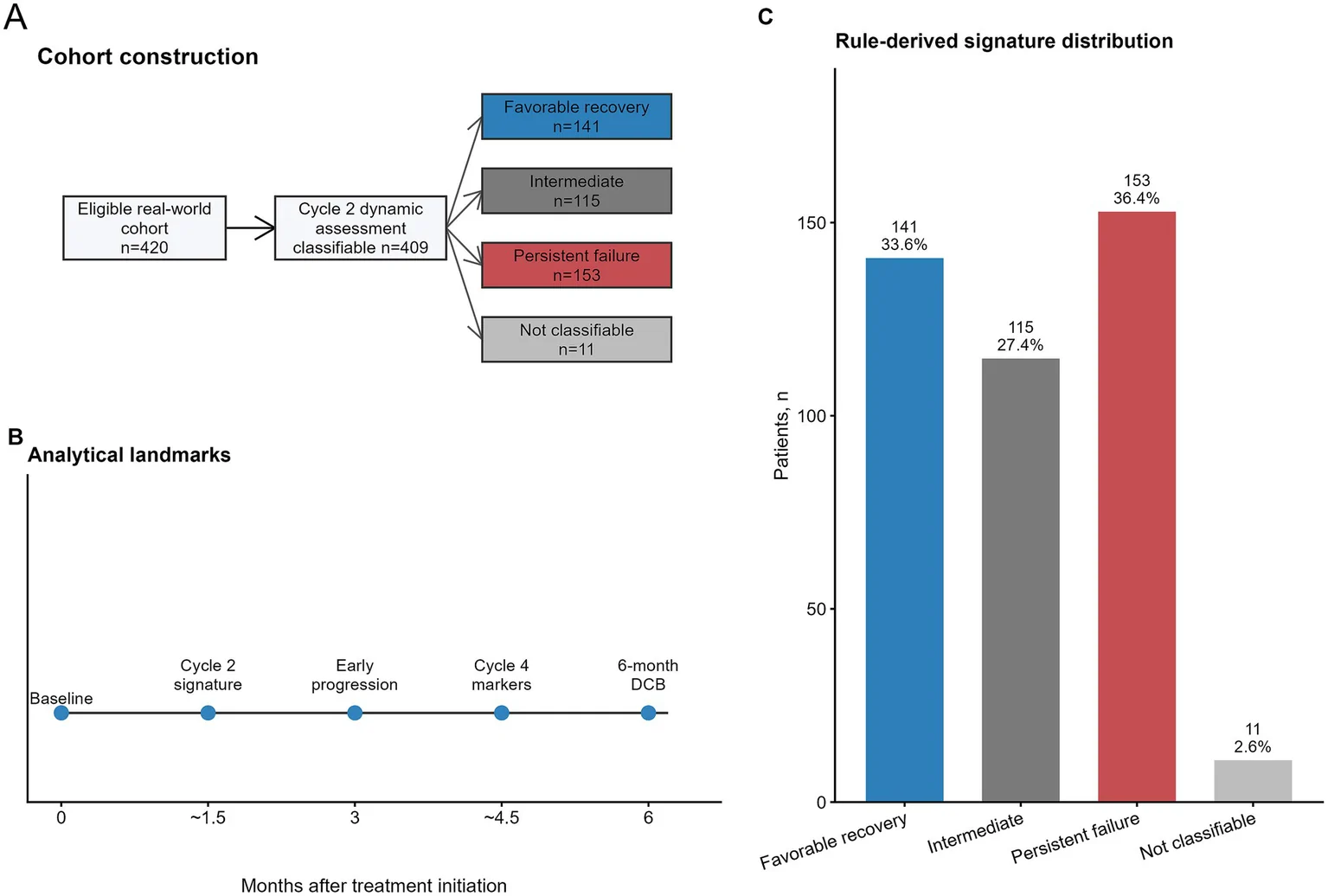
Cohort construction, cycle 2 dynamic recovery assessment, and analytical landmarks. Panel **(A)** shows the eligible real-world cohort, the classifiable rule-derived cycle 2 dynamic-signature cohort, and signature group counts. Panel **(B)** summarizes the baseline, cycle 2, cycle 4, 3-month early progression, and 6-month durable benefit landmarks. Panel **(C)** shows signature distribution in the full cohort.

The source cohort included 420 patients (Figure 1). Eleven patients lacked sufficient cycle 2 information for dynamic signature classification, leaving 409 patients in the primary classifiable cohort; the overall pattern of missing data is summarized in Supplementary Table S1 and Supplementary Figure S1. Among these classifiable patients, 141 (34.5%) were classified as favorable recovery, 115 (28.1%) as intermediate, and 153 (37.4%) as persistent failure.

Baseline characteristics are summarized in Table 1. The median age was 65 years, 285 patients (69.7%) were male, and 363 (88.8%) had stage IV disease. Adenocarcinoma was the most common histology (60.1%), followed by squamous cell carcinoma (33.0%). Most patients received ICI plus chemotherapy (72.1%), and PD-L1 TPS was unknown in 26.2%. Baseline NLR, PLR, SII, SIRI, PNI, ALI, and LIPI did not differ materially across the dynamic signature groups. Hypertension differed across groups (*p* = 0.047), whereas COPD, PD-L1 TPS category, actionable driver status, first-line treatment pattern, and antiangiogenic-containing treatment did not show statistically significant between-group differences.

**Table 1:** Baseline characteristics according to the rule-derived cycle 2 dynamic recovery signature.

Characteristic	Overall	Favorable recovery	Intermediate	Persistent failure	*p* value
Age, years	65 (59–71)	64 (59–70)	65 (60–71)	66 (59–72)	0.327
Sex					0.522
Male	285 (69.7)	98 (69.5)	76 (66.1)	111 (72.5)	
Female	124 (30.3)	43 (30.5)	39 (33.9)	42 (27.5)	
Body mass index, kg/m^2^	23.2 (21.0–25.1)	22.8 (20.9–24.9)	23.5 (21.0–25.2)	23.6 (21.0–25.3)	0.425
Smoking status					0.245
Never	160 (39.1)	51 (36.2)	51 (44.3)	58 (37.9)	
Former	134 (32.8)	52 (36.9)	38 (33.0)	44 (28.8)	
Current	115 (28.1)	38 (27.0)	26 (22.6)	51 (33.3)	
ECOG performance status	1 (1–1)	1 (1–1)	1 (0–1)	1 (1–1)	0.576
COPD					0.251
Yes	72 (17.6)	22 (15.6)	26 (22.6)	24 (15.7)	
Hypertension					0.047
Yes	52 (12.7)	10 (7.1)	18 (15.7)	24 (15.7)	
Diabetes					0.669
Yes	34 (8.3)	14 (9.9)	8 (7.0)	12 (7.8)	
Histology					0.876
Adenocarcinoma	246 (60.1)	88 (62.4)	71 (61.7)	87 (56.9)	
Squamous cell carcinoma	135 (33.0)	44 (31.2)	37 (32.2)	54 (35.3)	
NSCLC-NOS	28 (6.8)	9 (6.4)	7 (6.1)	12 (7.8)	
Disease stage					0.572
IV	363 (88.8)	123 (87.2)	105 (91.3)	135 (88.2)	
IIIB/IIIC unresectable or recurrent	46 (11.2)	18 (12.8)	10 (8.7)	18 (11.8)	
Number of metastatic sites	1 (1–2)	1 (1–2)	1 (1–2)	1 (1–2)	0.316
Brain metastasis					0.446
Yes	54 (13.2)	18 (12.8)	12 (10.4)	24 (15.7)	
Liver metastasis					0.051
Yes	62 (15.2)	14 (9.9)	24 (20.9)	24 (15.7)	
Bone metastasis					0.93
Yes	116 (28.4)	40 (28.4)	34 (29.6)	42 (27.5)	
Pleural metastasis					0.597
Yes	94 (23.0)	35 (24.8)	28 (24.3)	31 (20.3)	
PD-L1 TPS category					0.536
<1%	108 (26.4)	39 (27.7)	24 (20.9)	45 (29.4)	
1–49%	97 (23.7)	38 (27.0)	28 (24.3)	31 (20.3)	
> = 50%	97 (23.7)	33 (23.4)	29 (25.2)	35 (22.9)	
Unknown	107 (26.2)	31 (22.0)	34 (29.6)	42 (27.5)	
Actionable driver detected					0.141
Yes	10 (2.4)	2 (1.4)	1 (0.9)	7 (4.6)	
First-line treatment pattern					0.659
ICI + chemotherapy	295 (72.1)	95 (67.4)	86 (74.8)	114 (74.5)	
ICI monotherapy	56 (13.7)	25 (17.7)	15 (13.0)	16 (10.5)	
ICI + chemotherapy + antiangiogenic therapy	46 (11.2)	17 (12.1)	11 (9.6)	18 (11.8)	
ICI + antiangiogenic therapy	12 (2.9)	4 (2.8)	3 (2.6)	5 (3.3)	
PD-1/PD-L1 inhibitor					0.402
Sintilimab	124 (30.3)	45 (31.9)	37 (32.2)	42 (27.5)	
Pembrolizumab	92 (22.5)	38 (27.0)	23 (20.0)	31 (20.3)	
Camrelizumab	75 (18.3)	24 (17.0)	19 (16.5)	32 (20.9)	
Toripalimab	61 (14.9)	14 (9.9)	23 (20.0)	24 (15.7)	
Tislelizumab	50 (12.2)	19 (13.5)	11 (9.6)	20 (13.1)	
Atezolizumab	7 (1.7)	1 (0.7)	2 (1.7)	4 (2.6)	
Baseline NLR	3.33 (2.43–4.33)	3.33 (2.29–4.23)	3.37 (2.57–4.39)	3.22 (2.45–4.35)	0.725
Baseline PLR	192.2 (149.3–245.9)	194.8 (158.9–245.9)	183.6 (145.8–233.9)	193.2 (139.1–247.9)	0.53
Baseline SII	779 (562–1,102)	760 (569–1,054)	807 (581–1,143)	773 (523–1,114)	0.683
Baseline SIRI	1.64 (1.06–2.31)	1.60 (0.97–2.34)	1.73 (1.23–2.23)	1.60 (1.07–2.44)	0.834
Baseline PNI	45.8 (43.3–48.1)	46.1 (43.9–48.3)	45.9 (43.2–48.2)	45.1 (42.6–47.8)	0.07
Baseline ALI	273.6 (203.1–378.1)	276.9 (210.3–405.2)	265.2 (203.9–347.6)	286.7 (199.9–377.7)	0.568
Baseline LIPI					0.377
Good	312 (76.3)	106 (75.2)	84 (73.0)	122 (79.7)	
Intermediate	96 (23.5)	35 (24.8)	30 (26.1)	31 (20.3)	
Poor	1 (0.2)	0 (0.0)	1 (0.9)	0 (0.0)	

Data are median (IQR) or *n* (%). *p* values were calculated using Kruskal-Wallis tests for continuous variables and chi-square or Fisher exact tests for categorical variables. The primary classifiable cohort excluded 11 patients whose cycle 2 dynamic recovery signature was not classifiable. The rule-derived signature classified 141 patients as favorable recovery, 115 as intermediate, and 153 as persistent failure.

**Figure 2:**
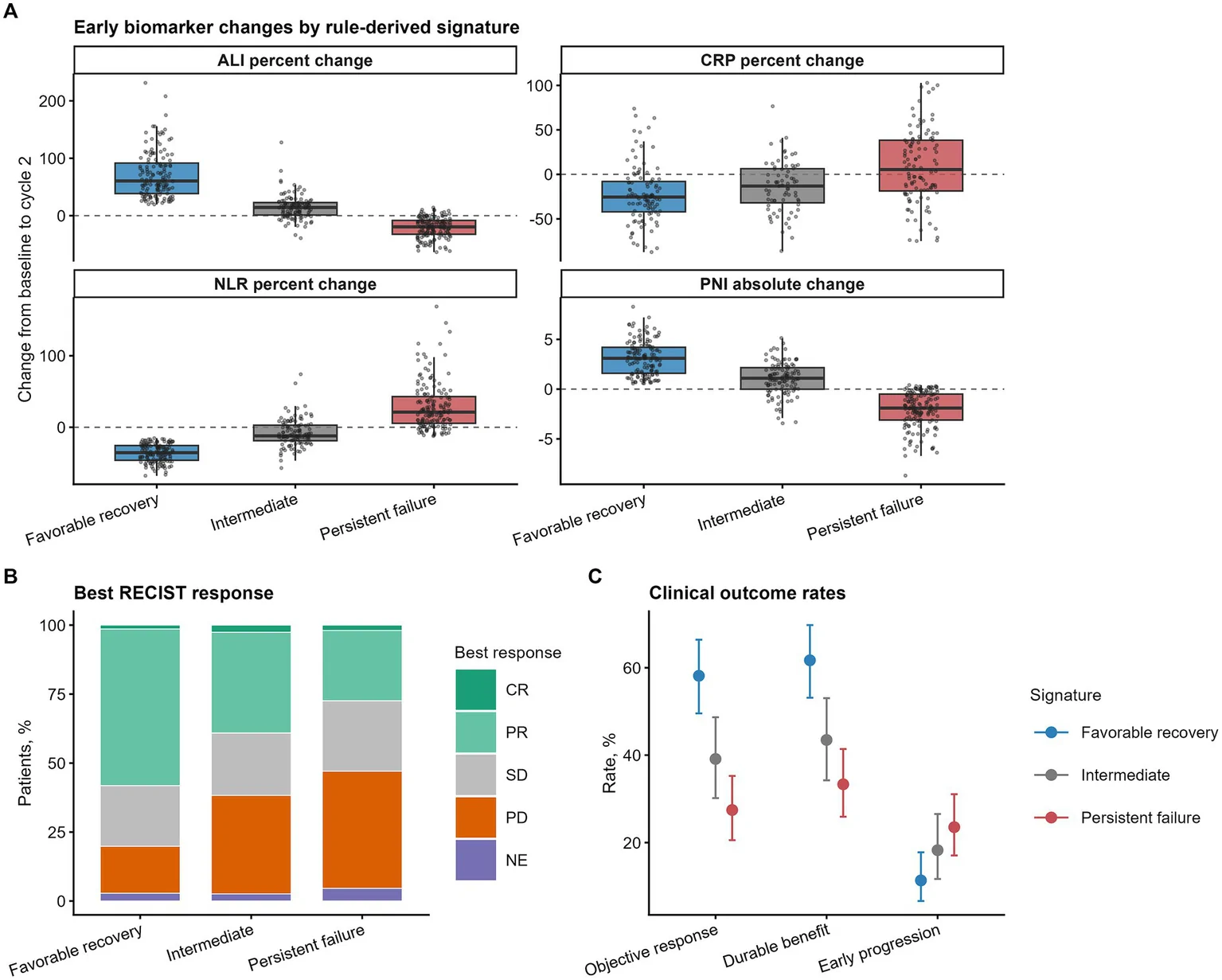
Early biomarker changes and clinical response according to the rule-derived dynamic signature. Panel **(A)** shows changes from baseline to cycle 2 in NLR, PNI, ALI, and CRP. Panel **(B)** shows best RECIST response composition. Panel **(C)** shows objective response, durable clinical benefit, and early progression rates with exact binomial 95% confidence intervals.

Early biomarker changes and clinical response are summarized in Figure 2, with longitudinal NLR, PNI, and ALI trajectories from baseline to cycle 2 and cycle 4 shown in Supplementary Figure S2. Clinical response differed substantially by dynamic signature (Table 2). Objective response was observed in 58.2% of patients with favorable recovery, 39.1% with an intermediate signature, and 27.5% with persistent failure (*p* < 0.001). Disease control followed the same pattern, occurring in 80.1, 61.7, and 52.9% of patients, respectively (p < 0.001). DCB at 6 months was achieved in 61.7% of the favorable recovery group, 43.5% of the intermediate group, and 33.3% of the persistent failure group (p < 0.001). Early progression within 3 months occurred in 11.3, 18.3, and 23.5% of patients, respectively (*p* = 0.024).

**Table 2:** Clinical efficacy, survival, and safety outcomes according to the rule-derived dynamic recovery signature.

Outcome	Overall	Favorable recovery	Intermediate	Persistent failure	*p* value
Best RECIST response					<0.001
CR	8 (2.0)	2 (1.4)	3 (2.6)	3 (2.0)	
PR	161 (39.4)	80 (56.7)	42 (36.5)	39 (25.5)	
SD	96 (23.5)	31 (22.0)	26 (22.6)	39 (25.5)	
PD	130 (31.8)	24 (17.0)	41 (35.7)	65 (42.5)	
NE	14 (3.4)	4 (2.8)	3 (2.6)	7 (4.6)	
Objective response	169 (41.3)	82 (58.2)	45 (39.1)	42 (27.5)	<0.001
Disease control	265 (64.8)	113 (80.1)	71 (61.7)	81 (52.9)	<0.001
Durable clinical benefit at 6 months	188 (46.0)	87 (61.7)	50 (43.5)	51 (33.3)	<0.001
Early progression within 3 months	73 (17.8)	16 (11.3)	21 (18.3)	36 (23.5)	0.024
Median PFS, months (95% CI)	12.0 (10.4–13.3)	20.8 (18.4–31.5)	11.6 (9.1–14.4)	7.0 (5.6–9.7)	<0.001
PFS event	260 (63.6)	69 (48.9)	76 (66.1)	115 (75.2)	<0.001
Median OS, months (95% CI)	19.6 (17.5–25.1)	27.6 (18.7–32.5)	15.3 (12.5–21.7)	19.8 (14.6–25.5)	0.057
Death	204 (49.9)	63 (44.7)	62 (53.9)	79 (51.6)	0.292
Any immune-related adverse event	88 (21.5)	30 (21.3)	27 (23.5)	31 (20.3)	0.815
Immune-related pneumonitis	25 (6.1)	5 (3.5)	10 (8.7)	10 (6.5)	0.223
Grade 3 or higher adverse event	61 (14.9)	22 (15.6)	14 (12.2)	25 (16.3)	0.613
Treatment discontinuation	52 (12.7)	16 (11.3)	15 (13.0)	21 (13.7)	0.823

Data are *n* (%) unless otherwise indicated. PFS and OS are reported as Kaplan–Meier median estimates with 95% confidence intervals. *p* values for PFS and OS are log-rank *p* values; other *p* values were calculated using chi-square or Fisher exact tests, as appropriate.

**Figure 3:**
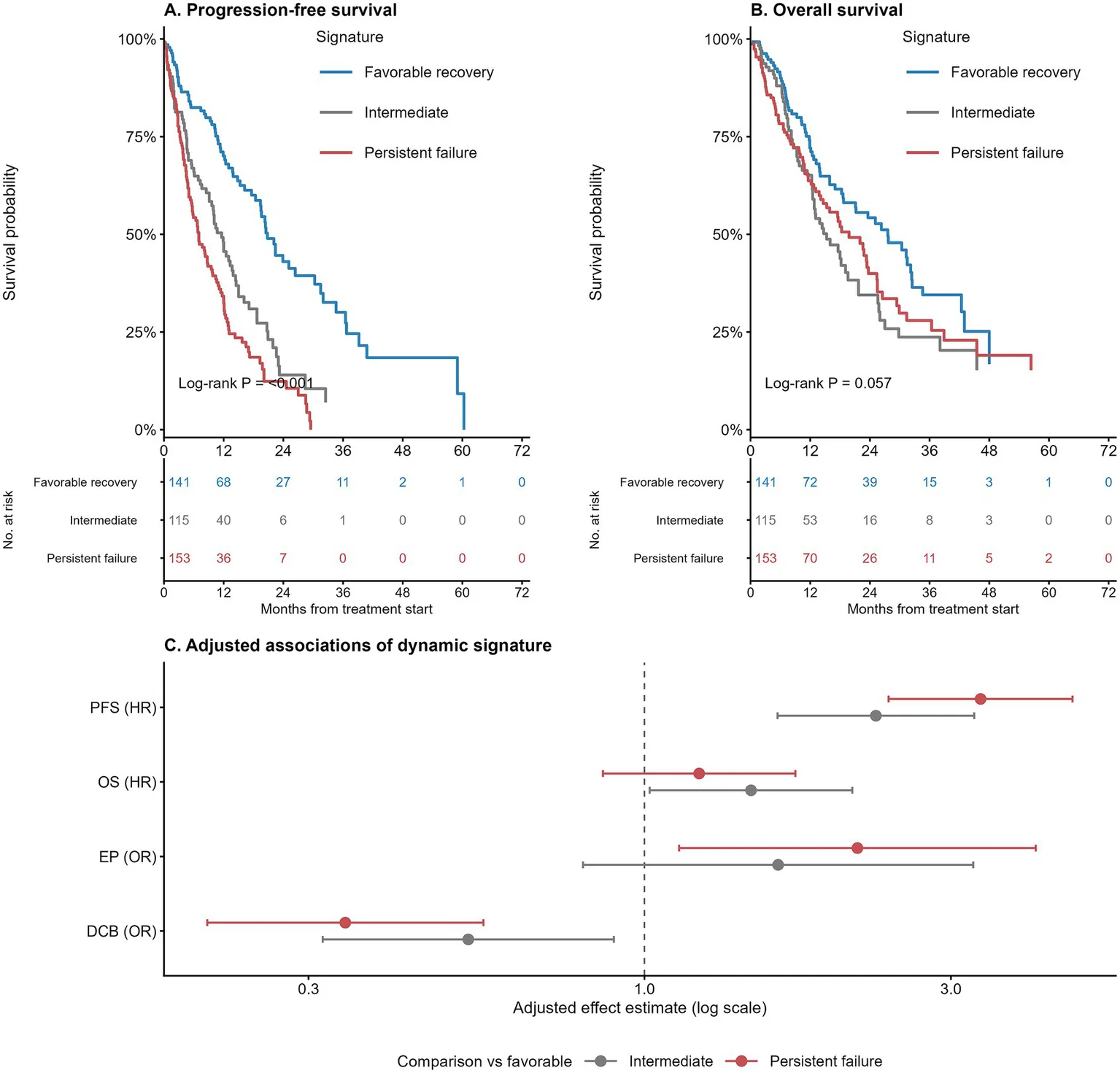
Survival outcomes and adjusted associations according to the rule-derived dynamic signature. Panels **(A,B)** show Kaplan–Meier curves for progression-free and overall survival, with log-rank *p* values and number-at-risk tables. Panel **(C)** shows multivariable-adjusted effect estimates for intermediate and persistent-failure signatures compared with favorable recovery.

Survival outcomes are shown in Figure 3. Median PFS was 20.8 months (95% CI, 18.4–31.5) in the favorable recovery group, 11.6 months (95% CI, 9.1–14.4) in the intermediate group, and 7.0 months (95% CI, 5.6–9.7) in the persistent failure group (log-rank *p* < 0.001). Median OS was 27.6 months (95% CI, 18.7–32.5), 15.3 months (95% CI, 12.5–21.7), and 19.8 months (95% CI, 14.6–25.5), respectively (log-rank *p* = 0.057).

After adjustment for clinical covariates and baseline NLR, PNI, and ALI, the dynamic signature remained associated with DCB, early progression, and PFS (Table 3). Compared with favorable recovery, persistent failure was associated with lower odds of DCB (adjusted OR, 0.34; 95% CI, 0.21–0.56; *p* < 0.001), higher odds of early progression (adjusted OR, 2.15; 95% CI, 1.13–4.07; *p* = 0.019), and higher risk of progression or death (adjusted HR for PFS, 3.34; 95% CI, 2.40–4.64; *p* < 0.001). The adjusted OS association was weaker: the intermediate group had higher mortality risk than favorable recovery (adjusted HR, 1.47; 95% CI, 1.02–2.11; *p* = 0.039), whereas persistent failure was not significantly associated with OS after adjustment (adjusted HR, 1.22; 95% CI, 0.86–1.72; *p* = 0.265). Global proportional hazards tests were not violated for either the PFS or OS model (Supplementary Table S4).

**Table 3:** Adjusted associations of the rule-derived dynamic recovery signature with clinical benefit and survival.

Endpoint	Comparison	Measure	Estimate	*p* value
Durable clinical benefit	Intermediate vs. Favorable recovery	Adjusted OR	0.53 (0.32–0.90)	0.018
Durable clinical benefit	Persistent failure vs. Favorable recovery	Adjusted OR	0.34 (0.21–0.56)	<0.001
Durable clinical benefit	Ordinal trend	OR per worse signature category	0.58 (0.46–0.75)	<0.001
Early progression	Intermediate vs. Favorable recovery	Adjusted OR	1.61 (0.80–3.25)	0.179
Early progression	Persistent failure vs. Favorable recovery	Adjusted OR	2.15 (1.13–4.07)	0.019
Early progression	Ordinal trend	OR per worse signature category	1.46 (1.06–1.99)	0.019
Progression-free survival	Intermediate vs. Favorable recovery	Adjusted HR	2.29 (1.61–3.26)	<0.001
Progression-free survival	Persistent failure vs. Favorable recovery	Adjusted HR	3.34 (2.40–4.64)	<0.001
Progression-free survival	Ordinal trend	HR per worse signature category	1.79 (1.53–2.10)	<0.001
Overall survival	Intermediate vs. Favorable recovery	Adjusted HR	1.47 (1.02–2.11)	0.039
Overall survival	Persistent failure vs. Favorable recovery	Adjusted HR	1.22 (0.86–1.72)	0.265
Overall survival	Ordinal trend	HR per worse signature category	1.10 (0.93–1.29)	0.284

Models adjusted for age, sex, ECOG performance status, histology, stage, number of metastatic sites, brain metastasis, liver metastasis, PD-L1 TPS category, first-line treatment pattern, and baseline NLR, PNI, and ALI. Binary endpoints used Firth-type penalized logistic regression; survival endpoints used Cox proportional hazards regression. Favorable recovery was the reference group.

Ordinal analyses supported graded associations for DCB, early progression, and PFS. Each step toward a worse signature category was associated with lower odds of DCB (OR, 0.58; 95% CI, 0.46–0.75), higher odds of early progression (OR, 1.46; 95% CI, 1.06–1.99), and shorter PFS (HR, 1.79; 95% CI, 1.53–2.10). The ordinal association with OS was not significant (HR, 1.10; 95% CI, 0.93–1.29). Individual component models were directionally consistent: increasing NLR percent change was associated with worse outcomes, whereas increasing PNI and ALI changes were associated with better outcomes (Supplementary Table S2 and Supplementary Figure S3). Primary associations for DCB and PFS were generally preserved in stage IV-only, non-monotherapy, and landmark sensitivity analyses (Supplementary Table S3).

**Figure 4:**
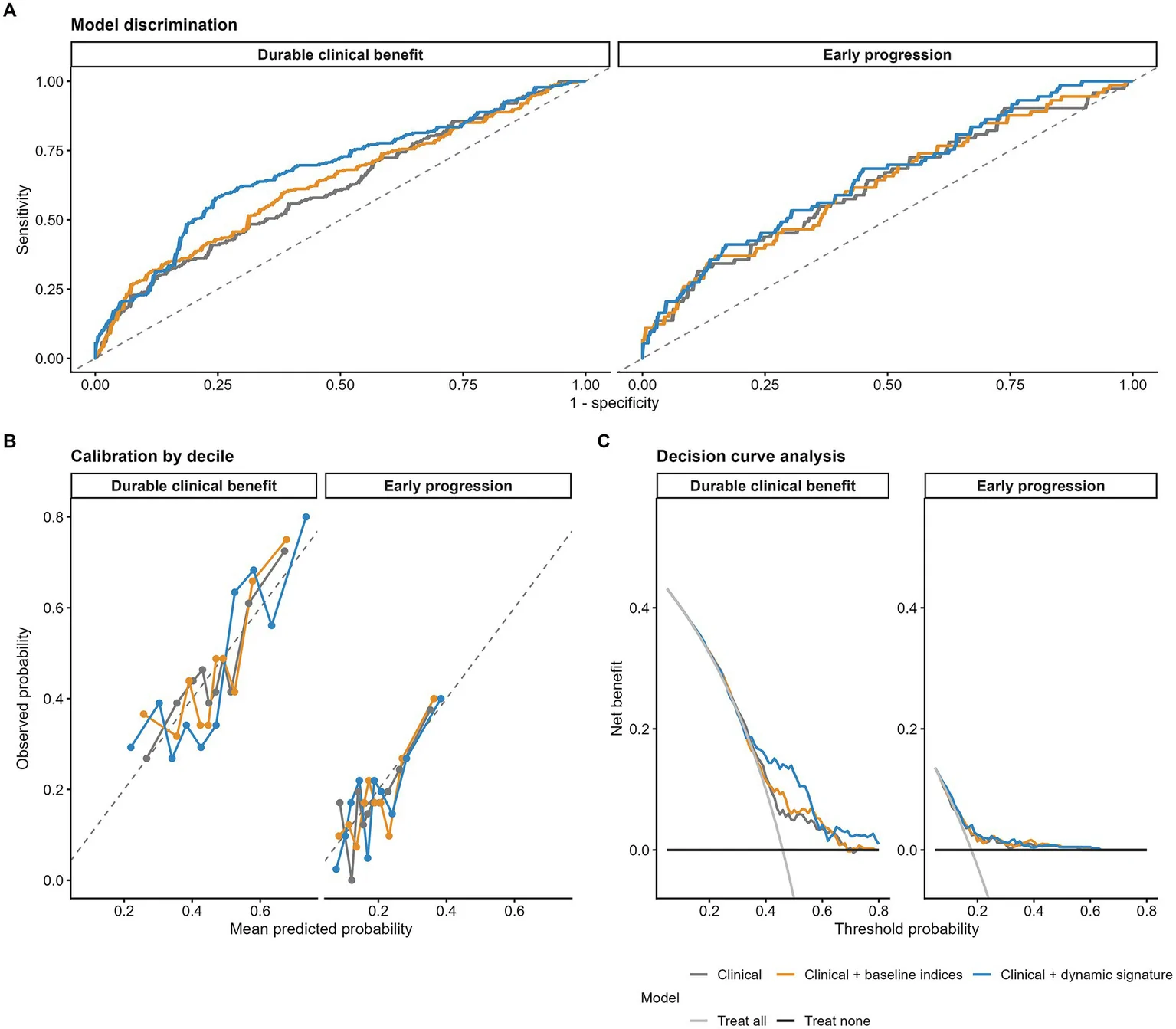
Predictive performance for durable clinical benefit and early progression. Panel **(A)** shows ROC curves for clinical, clinical plus baseline inflammatory/nutritional indices, and clinical plus dynamic signature models. Panel **(B)** shows apparent calibration by decile of predicted probability. Panel **(C)** shows decision curve analysis. Bootstrap optimism-corrected discrimination and calibration metrics are reported in Table 4.

Prediction analyses showed modest discrimination (Table 4; Figure 4). For DCB, the clinical model had an apparent AUC of 0.614 and an optimism-corrected AUC of 0.549; adding baseline inflammatory/nutritional indices resulted in an apparent AUC of 0.630 and corrected AUC of 0.553. Adding the dynamic signature increased the apparent AUC to 0.677 (95% CI, 0.625–0.730), with an optimism-corrected AUC of 0.619, corrected calibration slope of 0.71, and corrected Brier score of 0.243. For early progression, the apparent AUC of the clinical plus dynamic signature model was 0.654 (95% CI, 0.584–0.724), with an optimism-corrected AUC of 0.562 and corrected calibration slope of 0.57. These results indicate limited individual-level predictive accuracy, especially for early progression.

**Table 4:** Predictive performance for durable clinical benefit and early progression.

Endpoint	Model	Apparent AUC (95% CI)	Optimism-corrected AUC	Corrected calibration slope	Corrected Brier score	Bootstrap resamples
Durable clinical benefit	Clinical	0.614 (0.559–0.668)	0.549	0.61	0.253	200
Durable clinical benefit	Clinical + baseline indices	0.630 (0.575–0.684)	0.553	0.56	0.255	200
Durable clinical benefit	Clinical + dynamic signature	0.677 (0.625–0.730)	0.619	0.71	0.243	200
Early progression	Clinical	0.626 (0.553–0.700)	0.537	0.52	0.151	200
Early progression	Clinical + baseline indices	0.630 (0.557–0.703)	0.526	0.49	0.152	200
Early progression	Clinical + dynamic signature	0.654 (0.584–0.724)	0.562	0.57	0.151	200

AUC values are apparent estimates with DeLong 95% confidence intervals. Optimism-corrected AUC, calibration slope, and Brier score were estimated with 200 bootstrap resamples per model. Clinical variables included age, sex, ECOG performance status, histology, stage, number of metastatic sites, brain metastasis, liver metastasis, PD-L1 TPS category, and first-line treatment pattern.

Head-to-head analyses showed that the categorical composite signature did not outperform the individual continuous dynamic biomarkers in discrimination (Supplementary Table S5). For DCB, optimism-corrected AUCs were 0.642 for clinical plus dynamic NLR change, 0.640 for clinical plus dynamic PNI change, 0.633 for clinical plus dynamic ALI change, 0.651 for clinical plus all three dynamic components, and 0.618 for clinical plus the categorical composite signature. The apparent AUC of the composite signature was significantly lower than that of the model containing all three continuous dynamic components (AUC difference, −0.030; DeLong *p* = 0.013). Threshold sensitivity analyses showed directionally consistent associations for DCB and PFS across alternative rules (Supplementary Table S6).

Additional analyses addressed potential confounding and treatment heterogeneity (Supplementary Tables S7, S8). Associations with DCB and PFS were generally preserved after additional adjustment for hypertension and COPD, after excluding actionable driver alterations, after excluding unknown PD-L1 TPS, and after complete-case adjustment for baseline CRP. DCB and PFS associations were also preserved in the ICI plus chemotherapy subgroup and in patients not receiving antiangiogenic therapy. There was no statistical evidence of effect modification by PD-L1 TPS category, first-line treatment pattern, or antiangiogenic exposure, although subgroup power was limited, particularly for antiangiogenic-containing regimens.

Safety outcomes were not consistently aligned with the recovery signature (Supplementary Figure S4). Any irAE occurred in 21.5% of patients overall, with similar rates across the favorable, intermediate, and persistent failure groups (21.3, 23.5, and 20.3%; *p* = 0.815). Grade 3 or higher adverse events, treatment discontinuation, and immune-related pneumonitis did not differ significantly across groups.

## 4 Discussion

This real-world cohort study found that a rule-derived cycle 2 immune-inflammatory and nutritional recovery signature was associated with DCB and PFS in advanced NSCLC treated with first-line PD-1/PD-L1 inhibitor-based therapy. Persistent failure was associated with a lower probability of DCB, a higher probability of early progression, and shorter PFS after adjustment for clinical characteristics and baseline NLR, PNI, and ALI. In contrast, the adjusted OS association was weaker and not monotonic, and model discrimination was modest. These findings support the signature as an exploratory risk-stratification summary rather than a clinically ready prediction tool.

The biological rationale for evaluating early change is that a baseline blood test captures the pretreatment host-tumor state, whereas an on-treatment change also reflects early tumor control, inflammatory adaptation, nutritional reserve, and treatment tolerance. Decreasing NLR may indicate attenuation of neutrophil-driven inflammatory and immunosuppressive pressure, whereas preserved or increased lymphocyte reserve may reflect a more permissive antitumor immune environment (17, 18). Increasing PNI incorporates albumin and lymphocyte information and may reflect improved nutritional and immune reserve. Increasing ALI integrates body mass, albumin, and NLR, and therefore summarizes both inflammatory and nutritional recovery. The observed direction of each individual component was consistent with this framework.

The findings also fit with previous evidence on the individual components. A high pretreatment NLR has been associated with worse outcomes in nivolumab-treated advanced NSCLC and across meta-analyses of lung cancer immunotherapy cohorts (15, 16, 33, 34). LIPI and LDH/dNLR-based analyses have shown that combining inflammatory burden with tissue-injury markers can improve prognostic stratification during ICI therapy (19, 22). PNI has been associated with outcomes in ICI-treated lung cancer in systematic reviews (21, 35). ALI has been reported to predict early progression in nivolumab-treated advanced NSCLC, and a more recent study also supported ALI and GRIm score as ICI efficacy markers across lung and gastrointestinal cancers (20, 36).

The head-to-head prediction analyses clarify the role of the composite signature. The categorical signature did not outperform individual continuous dynamic markers or a model containing all three continuous components. This finding argues against interpreting the composite signature as statistically superior to its components. Its potential value is different: it provides a transparent, rule-based grouping that can summarize concordant recovery, partial recovery, and absent recovery without requiring optimized weights or complex calculations. Therefore, the composite signature should be viewed as an interpretable summary of biologically related host-response domains, not as a replacement for continuous biomarker modeling.

The relationship between the dynamic signature and established predictive biomarkers also requires cautious interpretation. PD-L1 TPS distribution did not differ significantly across signature groups, and interaction analyses did not show statistical evidence that the signature effect differed by PD-L1 category. This pattern suggests that the dynamic signature may capture host inflammatory and nutritional adaptation that is partly independent of PD-L1-driven tumor immune sensitivity. Similarly, associations with DCB and PFS were generally preserved across treatment-sensitivity analyses, and formal interaction tests did not support clear effect modification by treatment pattern or antiangiogenic exposure. These analyses reduce, but do not eliminate, concern about treatment-selection bias in this retrospective setting.

Infection-related confounding remains important. Antibiotic exposure around immune checkpoint inhibitor initiation has been associated with poorer outcomes in patients receiving PD-1/PD-L1 inhibitors, possibly through gut microbiome disruption and impaired antitumor immune function (37, 38). The present dataset did not systematically capture antibiotic type, route, duration, indication, baseline obstructive pneumonia, fever, or procalcitonin. COPD and baseline CRP were evaluated as partial proxy variables where available, and the DCB and PFS associations were generally preserved. Nevertheless, persistent inflammatory failure at cycle 2 should not be interpreted as treatment failure without clinical assessment for infection, obstructive pneumonia, treatment toxicity, or other reversible causes of systemic inflammation.

The DCB endpoint should also be interpreted clinically rather than biologically. DCB incorporates complete response, partial response, and stable disease lasting at least 6 months. Stable disease is heterogeneous and may include indolent tumor biology, delayed treatment effect, or competing non-tumor influences. For this reason, DCB was paired with early progression and time-to-event outcomes rather than treated as a stand-alone surrogate of immune sensitivity. The signature showed its strongest and most consistent signal for DCB and PFS; early-progression discrimination was limited, and OS was not robust after adjustment. The signature should therefore not be used as a stand-alone rule to identify early progression or to justify early treatment switching.

The safety findings support a similar distinction. Any irAE, grade 3 or higher adverse events, pneumonitis, and treatment discontinuation did not differ significantly across signature groups. Therefore, the current data support the signature primarily as an efficacy-related and disease-control marker, not as a toxicity marker. Inflammatory recovery and immune-related toxicity are not interchangeable biological processes.

Several strengths are worth noting. The study focused on first-line PD-1/PD-L1 inhibitor-based therapy, a setting directly relevant to current guidelines and supported by randomized first-line chemoimmunotherapy evidence (3, 4, 39). The exposure was assessed early during treatment, before the 3-month and 6-month outcome landmarks. The analyses adjusted for clinical covariates and baseline inflammatory/nutritional indices, examined individual components, tested alternative thresholds, evaluated confounding and treatment heterogeneity, and reported bootstrap optimism-corrected discrimination and calibration in line with prediction-model reporting principles (31, 32). Decision curve analysis helped separate statistical discrimination from potential clinical utility (30).

This study has limitations. First, it was a retrospective single-center study, so residual confounding, treatment-selection bias, and center-specific practice patterns cannot be excluded; prospective multicenter external validation is necessary. Second, 11 patients were not classifiable because of missing cycle 2 information, and other markers such as CRP and tumor markers had greater missingness. CRP was therefore evaluated in complete-case sensitivity analysis rather than incorporated into the primary composite signature. Third, the cycle 2 signature creates a landmark-like exposure; the 1.5-month landmark sensitivity analysis reduced but did not eliminate concerns about immortal-time and early-survivor bias. Fourth, PD-L1 TPS was unknown in a substantial proportion of patients, and the dataset did not support integrated analyses of TMB, genomic co-alterations, radiomics, or circulating tumor DNA. Fifth, antibiotic exposure, obstructive pneumonia, fever, procalcitonin, severe baseline infection, and pre-existing autoimmune disease were not systematically captured. Sixth, the threshold rules and the composite signature were internally assessed only; the cutoffs have not been externally validated. Seventh, no prospective interventional data show that signature-guided imaging intervals, treatment escalation, or treatment switching improves outcomes.

In summary, early recovery of immune-inflammatory and nutritional status was associated with DCB and PFS in advanced NSCLC receiving first-line PD-1/PD-L1 inhibitor-based therapy. The rule-derived signature showed only moderate or limited discriminative performance and was internally validated in a single-center retrospective cohort. Independent external cohort studies and prospective clinical trials are required before it can be adopted for clinical risk stratification, and it cannot be used alone to guide treatment adjustments.

## Data Availability

The original contributions presented in the study are included in the article/Supplementary material, further inquiries can be directed to the corresponding author.
