# Supplementary material for: Early dynamic immune-inflammatory and nutritional recovery signature is associated with durable benefit and early progression in advanced non-small cell lung cancer receiving first-line PD-1/PD-L1 inhibitor-based therapy: a real-world retrospective cohort study
Source: Front Med (Lausanne). 2026 Aug 14;13:1900721. doi: 10.3389/fmed.2026.1900721 (PMC13522164; doi:10.3389/fmed.2026.1900721)
Supplement: Supplementary file 1 [file Data_Sheet_1.docx]

**Supplementary File**

Supplementary tables and figure legends

# Supplementary Tables

**Supplementary Table S1. Missingness profile**

| **Variable** | **Missing** | **Missing, %** |
| --- | --- | --- |
| Cycle 4 CEA | 196 | 46.7 |
| Cycle 4 CRP | 172 | 41.0 |
| Cycle 4 CYFRA21-1 | 162 | 38.6 |
| CRP change, baseline to cycle 2 | 139 | 33.1 |
| Cycle 2 SCC antigen | 136 | 32.4 |
| Cycle 4 WBC count | 122 | 29.0 |
| Cycle 4 neutrophil count | 122 | 29.0 |
| Cycle 4 lymphocyte count | 122 | 29.0 |
| Cycle 4 platelet count | 122 | 29.0 |
| Cycle 4 albumin | 122 | 29.0 |
| Cycle 4 LDH | 122 | 29.0 |
| Cycle 4 NLR | 122 | 29.0 |
| Cycle 4 SII | 122 | 29.0 |
| Cycle 4 PNI | 122 | 29.0 |
| Cycle 4 ALI | 122 | 29.0 |
| Cycle 4 LIPI | 122 | 29.0 |
| PD-L1 TPS, % | 110 | 26.2 |
| Cycle 2 CEA | 98 | 23.3 |
| Cycle 2 CYFRA21-1 | 85 | 20.2 |
| Baseline SCC antigen | 84 | 20.0 |
| Cycle 2 CRP | 79 | 18.8 |
| Baseline CRP | 76 | 18.1 |
| Baseline CEA | 46 | 11.0 |
| Baseline CYFRA21-1 | 28 | 6.7 |
| Cycle 2 WBC count | 11 | 2.6 |
| Cycle 2 neutrophil count | 11 | 2.6 |
| Cycle 2 lymphocyte count | 11 | 2.6 |
| Cycle 2 monocyte count | 11 | 2.6 |
| Cycle 2 platelet count | 11 | 2.6 |
| Cycle 2 albumin | 11 | 2.6 |
| Cycle 2 LDH | 11 | 2.6 |
| Cycle 2 NLR | 11 | 2.6 |
| Cycle 2 PLR | 11 | 2.6 |
| Cycle 2 LMR | 11 | 2.6 |
| Cycle 2 SII | 11 | 2.6 |
| Cycle 2 SIRI | 11 | 2.6 |
| Cycle 2 PNI | 11 | 2.6 |
| Cycle 2 ALI | 11 | 2.6 |
| Cycle 2 dNLR | 11 | 2.6 |
| Cycle 2 LIPI | 11 | 2.6 |
| NLR change, baseline to cycle 2 | 11 | 2.6 |
| PNI change, baseline to cycle 2 | 11 | 2.6 |
| ALI change, baseline to cycle 2 | 11 | 2.6 |

Only variables with at least one missing value are shown. Missingness was calculated with the full cohort (n = 420) as the denominator.

**Supplementary Table S2. Adjusted associations of individual dynamic components**

| **Marker** | **Endpoint** | **Measure** | **Estimate** | **P value** |
| --- | --- | --- | --- | --- |
| NLR percent change per SD | Durable clinical benefit | Adjusted OR | 0.54 (0.42-0.69) | <0.001 |
| NLR percent change per SD | Early progression | Adjusted OR | 1.43 (1.12-1.83) | 0.004 |
| NLR percent change per SD | Progression-free survival | Adjusted HR | 1.64 (1.47-1.84) | <0.001 |
| NLR percent change per SD | Overall survival | Adjusted HR | 1.24 (1.07-1.45) | 0.005 |
| PNI absolute change per SD | Durable clinical benefit | Adjusted OR | 1.73 (1.38-2.17) | <0.001 |
| PNI absolute change per SD | Early progression | Adjusted OR | 0.76 (0.59-0.99) | 0.041 |
| PNI absolute change per SD | Progression-free survival | Adjusted HR | 0.63 (0.56-0.72) | <0.001 |
| PNI absolute change per SD | Overall survival | Adjusted HR | 0.86 (0.74-1.00) | 0.048 |
| ALI percent change per SD | Durable clinical benefit | Adjusted OR | 1.62 (1.30-2.02) | <0.001 |
| ALI percent change per SD | Early progression | Adjusted OR | 0.69 (0.51-0.92) | 0.012 |
| ALI percent change per SD | Progression-free survival | Adjusted HR | 0.56 (0.48-0.66) | <0.001 |
| ALI percent change per SD | Overall survival | Adjusted HR | 0.85 (0.72-0.99) | 0.040 |

Each dynamic marker was evaluated per one standard deviation change from baseline to cycle 2, adjusted for clinical covariates and baseline NLR, PNI, and ALI. Binary endpoints used Firth-type penalized logistic regression; survival endpoints used Cox proportional hazards regression.

**Supplementary Table S3. Sensitivity analyses for persistent failure versus favorable recovery**

| **Analysis** | **Endpoint** | **Measure** | **Estimate** | **P value** |
| --- | --- | --- | --- | --- |
| Primary classifiable cohort | Durable clinical benefit | Adjusted OR | 0.34 (0.21-0.56) | <0.001 |
| Primary classifiable cohort | Early progression | Adjusted OR | 2.15 (1.13-4.07) | 0.019 |
| Primary classifiable cohort | Progression-free survival | Adjusted HR | 3.34 (2.40-4.64) | <0.001 |
| Primary classifiable cohort | Overall survival | Adjusted HR | 1.22 (0.86-1.72) | 0.265 |
| Stage IV only | Durable clinical benefit | Adjusted OR | 0.32 (0.19-0.55) | <0.001 |
| Stage IV only | Early progression | Adjusted OR | 1.71 (0.88-3.32) | 0.114 |
| Stage IV only | Progression-free survival | Adjusted HR | 3.05 (2.17-4.29) | <0.001 |
| Stage IV only | Overall survival | Adjusted HR | 1.24 (0.86-1.79) | 0.254 |
| Excluding ICI monotherapy | Durable clinical benefit | Adjusted OR | 0.35 (0.21-0.60) | <0.001 |
| Excluding ICI monotherapy | Early progression | Adjusted OR | 2.21 (1.10-4.46) | 0.026 |
| Excluding ICI monotherapy | Progression-free survival | Adjusted HR | 3.12 (2.19-4.45) | <0.001 |
| Excluding ICI monotherapy | Overall survival | Adjusted HR | 1.13 (0.78-1.64) | 0.515 |
| 1.5-month landmark for survival endpoints | Durable clinical benefit | Adjusted OR | 0.33 (0.19-0.56) | <0.001 |
| 1.5-month landmark for survival endpoints | Early progression | Adjusted OR | 1.62 (0.73-3.61) | 0.237 |
| 1.5-month landmark for survival endpoints | Progression-free survival | Adjusted HR | 3.43 (2.41-4.89) | <0.001 |
| 1.5-month landmark for survival endpoints | Overall survival | Adjusted HR | 1.25 (0.87-1.78) | 0.225 |

Persistent failure was compared with favorable recovery. The 1.5-month landmark sensitivity analysis is intended to reduce immortal-time concerns for cycle 2 biomarker-based survival comparisons.

**Supplementary Table S4. Proportional hazards diagnostics**

| **Model** | **Term** | **Chi-square** | **P value** |
| --- | --- | --- | --- |
| PFS | age10 | 0.09 | 0.762 |
| PFS | sex | 0.92 | 0.338 |
| PFS | ecog_ps | 0.25 | 0.615 |
| PFS | histology | 0.36 | 0.837 |
| PFS | stage_iv | 4.63 | 0.031 |
| PFS | metastatic_sites_count | 0.3 | 0.585 |
| PFS | brain_metastasis | 2.21 | 0.137 |
| PFS | liver_metastasis | 0.09 | 0.764 |
| PFS | pd_l1_for_model | 4.05 | 0.256 |
| PFS | first_line_treatment_pattern | 1.05 | 0.79 |
| PFS | baseline_nlr_z | 0.33 | 0.566 |
| PFS | baseline_pni_z | 3.83 | 0.05 |
| PFS | baseline_ali_z | 0.31 | 0.577 |
| PFS | sig3 | 0.62 | 0.733 |
| PFS | GLOBAL | 25.27 | 0.191 |
| OS | age10 | 1.89 | 0.169 |
| OS | sex | 0.59 | 0.442 |
| OS | ecog_ps | 2.17 | 0.141 |
| OS | histology | 4.33 | 0.115 |
| OS | stage_iv | 8.99 | 0.003 |
| OS | metastatic_sites_count | 5.19 | 0.023 |
| OS | brain_metastasis | 0.39 | 0.535 |
| OS | liver_metastasis | 2.18 | 0.14 |
| OS | pd_l1_for_model | 3.59 | 0.309 |
| OS | first_line_treatment_pattern | 1.46 | 0.693 |
| OS | baseline_nlr_z | 0.22 | 0.637 |
| OS | baseline_pni_z | 0.31 | 0.579 |
| OS | baseline_ali_z | 0.28 | 0.598 |
| OS | sig3 | 3.63 | 0.163 |
| OS | GLOBAL | 27.82 | 0.114 |

Schoenfeld residual tests are shown for the multivariable Cox models.

**Supplementary Table S5. Head-to-head prediction performance of composite and individual dynamic biomarkers. Panel A. Model performance**

| **Endpoint** | **Model** | **Apparent AUC (95% CI)** | **Optimism-corrected AUC** | **Corrected calibration slope** | **Corrected Brier score** | **Bootstrap resamples** |
| --- | --- | --- | --- | --- | --- | --- |
| DCB | Clinical | 0.614 (0.559-0.668) | 0.546 | 0.59 | 0.253 | 200 |
| DCB | Clinical + baseline indices | 0.630 (0.575-0.684) | 0.551 | 0.56 | 0.256 | 200 |
| DCB | Clinical + dynamic NLR change | 0.694 (0.643-0.745) | 0.642 | 0.76 | 0.237 | 200 |
| DCB | Clinical + dynamic PNI change | 0.694 (0.643-0.745) | 0.64 | 0.74 | 0.238 | 200 |
| DCB | Clinical + dynamic ALI change | 0.689 (0.637-0.740) | 0.633 | 0.72 | 0.242 | 200 |
| DCB | Clinical + all dynamic components | 0.707 (0.657-0.757) | 0.651 | 0.74 | 0.236 | 200 |
| DCB | Clinical + composite signature | 0.677 (0.625-0.730) | 0.618 | 0.71 | 0.243 | 200 |
| DCB | Clinical + baseline indices + composite signature | 0.683 (0.630-0.735) | 0.619 | 0.69 | 0.244 | 200 |
| Early progression | Clinical | 0.626 (0.553-0.700) | 0.535 | 0.51 | 0.151 | 200 |
| Early progression | Clinical + baseline indices | 0.630 (0.557-0.703) | 0.526 | 0.48 | 0.154 | 200 |
| Early progression | Clinical + dynamic NLR change | 0.662 (0.593-0.732) | 0.57 | 0.58 | 0.152 | 200 |
| Early progression | Clinical + dynamic PNI change | 0.643 (0.572-0.713) | 0.551 | 0.56 | 0.151 | 200 |
| Early progression | Clinical + dynamic ALI change | 0.658 (0.587-0.730) | 0.57 | 0.59 | 0.151 | 200 |
| Early progression | Clinical + all dynamic components | 0.663 (0.593-0.732) | 0.565 | 0.56 | 0.152 | 200 |
| Early progression | Clinical + composite signature | 0.654 (0.584-0.724) | 0.563 | 0.56 | 0.151 | 200 |
| Early progression | Clinical + baseline indices + composite signature | 0.658 (0.589-0.728) | 0.555 | 0.54 | 0.153 | 200 |

AUC values are apparent estimates with DeLong 95% confidence intervals. Optimism-corrected AUC, calibration slope, and Brier score were estimated using 200 bootstrap resamples. The composite signature model was evaluated as a clinically interpretable categorical summary rather than as a statistically optimized replacement for individual continuous dynamic markers.

**Supplementary Table S5. Panel B. Apparent AUC contrasts for the composite signature model**

| **Endpoint** | **Comparison** | **Composite apparent AUC** | **Comparator apparent AUC** | **AUC difference** | **DeLong P value** |
| --- | --- | --- | --- | --- | --- |
| Durable clinical benefit | Composite signature vs Clinical + dynamic NLR change | 0.677 | 0.694 | -0.017 | 0.189 |
| Durable clinical benefit | Composite signature vs Clinical + dynamic PNI change | 0.677 | 0.694 | -0.017 | 0.232 |
| Durable clinical benefit | Composite signature vs Clinical + dynamic ALI change | 0.677 | 0.689 | -0.011 | 0.319 |
| Durable clinical benefit | Composite signature vs Clinical + all dynamic components | 0.677 | 0.707 | -0.03 | 0.013 |
| Early progression | Composite signature vs Clinical + dynamic NLR change | 0.654 | 0.662 | -0.008 | 0.595 |
| Early progression | Composite signature vs Clinical + dynamic PNI change | 0.654 | 0.643 | 0.011 | 0.422 |
| Early progression | Composite signature vs Clinical + dynamic ALI change | 0.654 | 0.658 | -0.004 | 0.759 |
| Early progression | Composite signature vs Clinical + all dynamic components | 0.654 | 0.663 | -0.008 | 0.572 |

AUC difference was calculated as composite apparent AUC minus comparator apparent AUC. DeLong P values compare apparent AUCs.

**Supplementary Table S6. Threshold sensitivity analyses for the rule-derived dynamic recovery signature. Panel A. Group distribution and crude outcome rates**

| **Scenario** | **Signature category** | **n** | **DCB (%)** | **Early progression (%)** | **PFS event (%)** | **Death (%)** |
| --- | --- | --- | --- | --- | --- | --- |
| Primary rule: 15/0.5/15 | Favorable recovery | 141 | 61.7 | 11.3 | 48.9 | 44.7 |
| Primary rule: 15/0.5/15 | Intermediate | 115 | 43.5 | 18.3 | 66.1 | 53.9 |
| Primary rule: 15/0.5/15 | Persistent failure | 153 | 33.3 | 23.5 | 75.2 | 51.6 |
| Less stringent: 10/0/10 | Favorable recovery | 176 | 59.1 | 11.4 | 50.0 | 46.6 |
| Less stringent: 10/0/10 | Intermediate | 102 | 41.2 | 22.5 | 68.6 | 52.9 |
| Less stringent: 10/0/10 | Persistent failure | 131 | 32.1 | 22.9 | 77.9 | 51.9 |
| More stringent: 20/1/20 | Favorable recovery | 113 | 62.8 | 10.6 | 47.8 | 45.1 |
| More stringent: 20/1/20 | Intermediate | 118 | 50.0 | 16.1 | 61.0 | 50.8 |
| More stringent: 20/1/20 | Persistent failure | 178 | 32.6 | 23.6 | 75.3 | 52.2 |
| PNI neutral: 15/0/15 | Favorable recovery | 151 | 60.9 | 10.6 | 49.7 | 45.7 |
| PNI neutral: 15/0/15 | Intermediate | 124 | 42.7 | 20.2 | 65.3 | 52.4 |
| PNI neutral: 15/0/15 | Persistent failure | 134 | 32.1 | 23.9 | 77.6 | 52.2 |
| NLR/ALI 20 with PNI 0.5 | Favorable recovery | 125 | 63.2 | 10.4 | 48.8 | 45.6 |
| NLR/ALI 20 with PNI 0.5 | Intermediate | 124 | 44.4 | 18.5 | 62.9 | 51.6 |
| NLR/ALI 20 with PNI 0.5 | Persistent failure | 160 | 33.8 | 23.1 | 75.6 | 51.9 |

The primary rule used NLR decrease >=15%, PNI increase >=0.5 points, and ALI increase >=15% to define improvement in each domain.

**Supplementary Table S6. Panel B. Adjusted associations under alternative threshold rules**

| **Scenario** | **n used** | **Endpoint** | **Comparison** | **Measure** | **Estimate** | **P value** |
| --- | --- | --- | --- | --- | --- | --- |
| Primary rule: 15/0.5/15 | 409 | Durable clinical benefit | Intermediate vs Favorable recovery | Adjusted OR | 0.53 (0.32-0.90) | 0.018 |
| Primary rule: 15/0.5/15 | 409 | Durable clinical benefit | Persistent failure vs Favorable recovery | Adjusted OR | 0.34 (0.21-0.56) | <0.001 |
| Primary rule: 15/0.5/15 | 409 | Early progression | Intermediate vs Favorable recovery | Adjusted OR | 1.61 (0.80-3.25) | 0.179 |
| Primary rule: 15/0.5/15 | 409 | Early progression | Persistent failure vs Favorable recovery | Adjusted OR | 2.15 (1.13-4.07) | 0.019 |
| Primary rule: 15/0.5/15 | 409 | Progression-free survival | Intermediate vs Favorable recovery | Adjusted HR | 2.29 (1.61-3.26) | <0.001 |
| Primary rule: 15/0.5/15 | 409 | Progression-free survival | Persistent failure vs Favorable recovery | Adjusted HR | 3.34 (2.40-4.64) | <0.001 |
| Primary rule: 15/0.5/15 | 409 | Overall survival | Intermediate vs Favorable recovery | Adjusted HR | 1.47 (1.02-2.11) | 0.039 |
| Primary rule: 15/0.5/15 | 409 | Overall survival | Persistent failure vs Favorable recovery | Adjusted HR | 1.22 (0.86-1.72) | 0.265 |
| Less stringent: 10/0/10 | 409 | Durable clinical benefit | Intermediate vs Favorable recovery | Adjusted OR | 0.56 (0.33-0.93) | 0.026 |
| Less stringent: 10/0/10 | 409 | Durable clinical benefit | Persistent failure vs Favorable recovery | Adjusted OR | 0.36 (0.22-0.59) | <0.001 |
| Less stringent: 10/0/10 | 409 | Early progression | Intermediate vs Favorable recovery | Adjusted OR | 2.02 (1.04-3.92) | 0.037 |
| Less stringent: 10/0/10 | 409 | Early progression | Persistent failure vs Favorable recovery | Adjusted OR | 2.01 (1.08-3.75) | 0.028 |
| Less stringent: 10/0/10 | 409 | Progression-free survival | Intermediate vs Favorable recovery | Adjusted HR | 2.46 (1.75-3.46) | <0.001 |
| Less stringent: 10/0/10 | 409 | Progression-free survival | Persistent failure vs Favorable recovery | Adjusted HR | 3.10 (2.27-4.24) | <0.001 |
| Less stringent: 10/0/10 | 409 | Overall survival | Intermediate vs Favorable recovery | Adjusted HR | 1.28 (0.90-1.82) | 0.175 |
| Less stringent: 10/0/10 | 409 | Overall survival | Persistent failure vs Favorable recovery | Adjusted HR | 1.08 (0.77-1.51) | 0.665 |
| More stringent: 20/1/20 | 409 | Durable clinical benefit | Intermediate vs Favorable recovery | Adjusted OR | 0.60 (0.35-1.05) | 0.074 |
| More stringent: 20/1/20 | 409 | Durable clinical benefit | Persistent failure vs Favorable recovery | Adjusted OR | 0.31 (0.18-0.51) | <0.001 |
| More stringent: 20/1/20 | 409 | Early progression | Intermediate vs Favorable recovery | Adjusted OR | 1.61 (0.75-3.46) | 0.222 |
| More stringent: 20/1/20 | 409 | Early progression | Persistent failure vs Favorable recovery | Adjusted OR | 2.32 (1.17-4.61) | 0.016 |
| More stringent: 20/1/20 | 409 | Progression-free survival | Intermediate vs Favorable recovery | Adjusted HR | 2.21 (1.49-3.26) | <0.001 |
| More stringent: 20/1/20 | 409 | Progression-free survival | Persistent failure vs Favorable recovery | Adjusted HR | 3.89 (2.73-5.56) | <0.001 |
| More stringent: 20/1/20 | 409 | Overall survival | Intermediate vs Favorable recovery | Adjusted HR | 1.25 (0.84-1.86) | 0.262 |
| More stringent: 20/1/20 | 409 | Overall survival | Persistent failure vs Favorable recovery | Adjusted HR | 1.20 (0.84-1.72) | 0.314 |
| PNI neutral: 15/0/15 | 409 | Durable clinical benefit | Intermediate vs Favorable recovery | Adjusted OR | 0.52 (0.32-0.87) | 0.012 |
| PNI neutral: 15/0/15 | 409 | Durable clinical benefit | Persistent failure vs Favorable recovery | Adjusted OR | 0.33 (0.20-0.55) | <0.001 |
| PNI neutral: 15/0/15 | 409 | Early progression | Intermediate vs Favorable recovery | Adjusted OR | 1.94 (0.99-3.80) | 0.054 |
| PNI neutral: 15/0/15 | 409 | Early progression | Persistent failure vs Favorable recovery | Adjusted OR | 2.28 (1.19-4.38) | 0.013 |
| PNI neutral: 15/0/15 | 409 | Progression-free survival | Intermediate vs Favorable recovery | Adjusted HR | 2.38 (1.70-3.34) | <0.001 |
| PNI neutral: 15/0/15 | 409 | Progression-free survival | Persistent failure vs Favorable recovery | Adjusted HR | 3.27 (2.36-4.53) | <0.001 |
| PNI neutral: 15/0/15 | 409 | Overall survival | Intermediate vs Favorable recovery | Adjusted HR | 1.36 (0.96-1.92) | 0.086 |
| PNI neutral: 15/0/15 | 409 | Overall survival | Persistent failure vs Favorable recovery | Adjusted HR | 1.15 (0.81-1.62) | 0.439 |
| NLR/ALI 20 with PNI 0.5 | 409 | Durable clinical benefit | Intermediate vs Favorable recovery | Adjusted OR | 0.49 (0.29-0.83) | 0.008 |
| NLR/ALI 20 with PNI 0.5 | 409 | Durable clinical benefit | Persistent failure vs Favorable recovery | Adjusted OR | 0.32 (0.19-0.54) | <0.001 |
| NLR/ALI 20 with PNI 0.5 | 409 | Early progression | Intermediate vs Favorable recovery | Adjusted OR | 1.87 (0.91-3.84) | 0.090 |
| NLR/ALI 20 with PNI 0.5 | 409 | Early progression | Persistent failure vs Favorable recovery | Adjusted OR | 2.27 (1.16-4.48) | 0.017 |
| NLR/ALI 20 with PNI 0.5 | 409 | Progression-free survival | Intermediate vs Favorable recovery | Adjusted HR | 2.41 (1.67-3.49) | <0.001 |
| NLR/ALI 20 with PNI 0.5 | 409 | Progression-free survival | Persistent failure vs Favorable recovery | Adjusted HR | 3.62 (2.56-5.10) | <0.001 |
| NLR/ALI 20 with PNI 0.5 | 409 | Overall survival | Intermediate vs Favorable recovery | Adjusted HR | 1.29 (0.89-1.87) | 0.179 |
| NLR/ALI 20 with PNI 0.5 | 409 | Overall survival | Persistent failure vs Favorable recovery | Adjusted HR | 1.21 (0.85-1.72) | 0.295 |

Models used the same covariate structure as the primary analyses. Favorable recovery was the reference group.

**Supplementary Table S7. Confounding and treatment-heterogeneity sensitivity analyses. Panel A. Additional adjustment and exclusion analyses**

| **Analysis** | **n used** | **Endpoint** | **Comparison** | **Measure** | **Estimate** | **P value** |
| --- | --- | --- | --- | --- | --- | --- |
| Primary rule-derived model | 409 | Durable clinical benefit | Intermediate vs Favorable recovery | Adjusted OR | 0.53 (0.32-0.90) | 0.018 |
| Primary rule-derived model | 409 | Durable clinical benefit | Persistent failure vs Favorable recovery | Adjusted OR | 0.34 (0.21-0.56) | <0.001 |
| Primary rule-derived model | 409 | Early progression | Intermediate vs Favorable recovery | Adjusted OR | 1.61 (0.80-3.25) | 0.179 |
| Primary rule-derived model | 409 | Early progression | Persistent failure vs Favorable recovery | Adjusted OR | 2.15 (1.13-4.07) | 0.019 |
| Primary rule-derived model | 409 | Progression-free survival | Intermediate vs Favorable recovery | Adjusted HR | 2.29 (1.61-3.26) | <0.001 |
| Primary rule-derived model | 409 | Progression-free survival | Persistent failure vs Favorable recovery | Adjusted HR | 3.34 (2.40-4.64) | <0.001 |
| Primary rule-derived model | 409 | Overall survival | Intermediate vs Favorable recovery | Adjusted HR | 1.47 (1.02-2.11) | 0.039 |
| Primary rule-derived model | 409 | Overall survival | Persistent failure vs Favorable recovery | Adjusted HR | 1.22 (0.86-1.72) | 0.265 |
| Additional adjustment for hypertension | 409 | Durable clinical benefit | Intermediate vs Favorable recovery | Adjusted OR | 0.54 (0.32-0.91) | 0.021 |
| Additional adjustment for hypertension | 409 | Durable clinical benefit | Persistent failure vs Favorable recovery | Adjusted OR | 0.35 (0.21-0.57) | <0.001 |
| Additional adjustment for hypertension | 409 | Early progression | Intermediate vs Favorable recovery | Adjusted OR | 1.69 (0.83-3.41) | 0.145 |
| Additional adjustment for hypertension | 409 | Early progression | Persistent failure vs Favorable recovery | Adjusted OR | 2.25 (1.18-4.29) | 0.013 |
| Additional adjustment for hypertension | 409 | Progression-free survival | Intermediate vs Favorable recovery | Adjusted HR | 2.28 (1.60-3.24) | <0.001 |
| Additional adjustment for hypertension | 409 | Progression-free survival | Persistent failure vs Favorable recovery | Adjusted HR | 3.32 (2.39-4.62) | <0.001 |
| Additional adjustment for hypertension | 409 | Overall survival | Intermediate vs Favorable recovery | Adjusted HR | 1.42 (0.99-2.05) | 0.058 |
| Additional adjustment for hypertension | 409 | Overall survival | Persistent failure vs Favorable recovery | Adjusted HR | 1.21 (0.86-1.71) | 0.282 |
| Additional adjustment for COPD | 409 | Durable clinical benefit | Intermediate vs Favorable recovery | Adjusted OR | 0.53 (0.32-0.90) | 0.019 |
| Additional adjustment for COPD | 409 | Durable clinical benefit | Persistent failure vs Favorable recovery | Adjusted OR | 0.34 (0.21-0.56) | <0.001 |
| Additional adjustment for COPD | 409 | Early progression | Intermediate vs Favorable recovery | Adjusted OR | 1.58 (0.79-3.18) | 0.200 |
| Additional adjustment for COPD | 409 | Early progression | Persistent failure vs Favorable recovery | Adjusted OR | 2.16 (1.14-4.10) | 0.018 |
| Additional adjustment for COPD | 409 | Progression-free survival | Intermediate vs Favorable recovery | Adjusted HR | 2.28 (1.60-3.24) | <0.001 |
| Additional adjustment for COPD | 409 | Progression-free survival | Persistent failure vs Favorable recovery | Adjusted HR | 3.34 (2.40-4.65) | <0.001 |
| Additional adjustment for COPD | 409 | Overall survival | Intermediate vs Favorable recovery | Adjusted HR | 1.46 (1.02-2.10) | 0.041 |
| Additional adjustment for COPD | 409 | Overall survival | Persistent failure vs Favorable recovery | Adjusted HR | 1.22 (0.86-1.72) | 0.259 |
| Additional adjustment for hypertension and COPD | 409 | Durable clinical benefit | Intermediate vs Favorable recovery | Adjusted OR | 0.54 (0.32-0.92) | 0.022 |
| Additional adjustment for hypertension and COPD | 409 | Durable clinical benefit | Persistent failure vs Favorable recovery | Adjusted OR | 0.35 (0.21-0.57) | <0.001 |
| Additional adjustment for hypertension and COPD | 409 | Early progression | Intermediate vs Favorable recovery | Adjusted OR | 1.65 (0.82-3.33) | 0.164 |
| Additional adjustment for hypertension and COPD | 409 | Early progression | Persistent failure vs Favorable recovery | Adjusted OR | 2.28 (1.19-4.34) | 0.012 |
| Additional adjustment for hypertension and COPD | 409 | Progression-free survival | Intermediate vs Favorable recovery | Adjusted HR | 2.26 (1.59-3.23) | <0.001 |
| Additional adjustment for hypertension and COPD | 409 | Progression-free survival | Persistent failure vs Favorable recovery | Adjusted HR | 3.33 (2.39-4.63) | <0.001 |
| Additional adjustment for hypertension and COPD | 409 | Overall survival | Intermediate vs Favorable recovery | Adjusted HR | 1.42 (0.98-2.05) | 0.062 |
| Additional adjustment for hypertension and COPD | 409 | Overall survival | Persistent failure vs Favorable recovery | Adjusted HR | 1.21 (0.86-1.71) | 0.275 |
| Excluding actionable drivers | 399 | Durable clinical benefit | Intermediate vs Favorable recovery | Adjusted OR | 0.52 (0.30-0.87) | 0.014 |
| Excluding actionable drivers | 399 | Durable clinical benefit | Persistent failure vs Favorable recovery | Adjusted OR | 0.36 (0.22-0.60) | <0.001 |
| Excluding actionable drivers | 399 | Early progression | Intermediate vs Favorable recovery | Adjusted OR | 1.60 (0.79-3.23) | 0.189 |
| Excluding actionable drivers | 399 | Early progression | Persistent failure vs Favorable recovery | Adjusted OR | 2.06 (1.08-3.94) | 0.028 |
| Excluding actionable drivers | 399 | Progression-free survival | Intermediate vs Favorable recovery | Adjusted HR | 2.39 (1.67-3.42) | <0.001 |
| Excluding actionable drivers | 399 | Progression-free survival | Persistent failure vs Favorable recovery | Adjusted HR | 3.42 (2.44-4.78) | <0.001 |
| Excluding actionable drivers | 399 | Overall survival | Intermediate vs Favorable recovery | Adjusted HR | 1.52 (1.06-2.20) | 0.024 |
| Excluding actionable drivers | 399 | Overall survival | Persistent failure vs Favorable recovery | Adjusted HR | 1.20 (0.85-1.71) | 0.301 |
| Excluding PD-L1 unknown | 302 | Durable clinical benefit | Intermediate vs Favorable recovery | Adjusted OR | 0.41 (0.22-0.76) | 0.004 |
| Excluding PD-L1 unknown | 302 | Durable clinical benefit | Persistent failure vs Favorable recovery | Adjusted OR | 0.34 (0.20-0.61) | <0.001 |
| Excluding PD-L1 unknown | 302 | Early progression | Intermediate vs Favorable recovery | Adjusted OR | 2.12 (0.91-4.91) | 0.081 |
| Excluding PD-L1 unknown | 302 | Early progression | Persistent failure vs Favorable recovery | Adjusted OR | 2.59 (1.20-5.59) | 0.015 |
| Excluding PD-L1 unknown | 302 | Progression-free survival | Intermediate vs Favorable recovery | Adjusted HR | 3.71 (2.40-5.73) | <0.001 |
| Excluding PD-L1 unknown | 302 | Progression-free survival | Persistent failure vs Favorable recovery | Adjusted HR | 4.25 (2.85-6.34) | <0.001 |
| Excluding PD-L1 unknown | 302 | Overall survival | Intermediate vs Favorable recovery | Adjusted HR | 1.23 (0.80-1.92) | 0.349 |
| Excluding PD-L1 unknown | 302 | Overall survival | Persistent failure vs Favorable recovery | Adjusted HR | 1.19 (0.79-1.79) | 0.417 |
| Complete-case adjustment for baseline CRP | 335 | Durable clinical benefit | Intermediate vs Favorable recovery | Adjusted OR | 0.56 (0.31-1.01) | 0.054 |
| Complete-case adjustment for baseline CRP | 335 | Durable clinical benefit | Persistent failure vs Favorable recovery | Adjusted OR | 0.39 (0.22-0.66) | <0.001 |
| Complete-case adjustment for baseline CRP | 335 | Early progression | Intermediate vs Favorable recovery | Adjusted OR | 1.86 (0.84-4.12) | 0.128 |
| Complete-case adjustment for baseline CRP | 335 | Early progression | Persistent failure vs Favorable recovery | Adjusted OR | 2.41 (1.18-4.91) | 0.015 |
| Complete-case adjustment for baseline CRP | 335 | Progression-free survival | Intermediate vs Favorable recovery | Adjusted HR | 2.22 (1.49-3.30) | <0.001 |
| Complete-case adjustment for baseline CRP | 335 | Progression-free survival | Persistent failure vs Favorable recovery | Adjusted HR | 3.15 (2.20-4.52) | <0.001 |
| Complete-case adjustment for baseline CRP | 335 | Overall survival | Intermediate vs Favorable recovery | Adjusted HR | 1.40 (0.93-2.10) | 0.108 |
| Complete-case adjustment for baseline CRP | 335 | Overall survival | Persistent failure vs Favorable recovery | Adjusted HR | 1.10 (0.76-1.60) | 0.617 |
| Complete-case adjustment for baseline CRP and COPD | 335 | Durable clinical benefit | Intermediate vs Favorable recovery | Adjusted OR | 0.57 (0.31-1.02) | 0.059 |
| Complete-case adjustment for baseline CRP and COPD | 335 | Durable clinical benefit | Persistent failure vs Favorable recovery | Adjusted OR | 0.39 (0.22-0.66) | <0.001 |
| Complete-case adjustment for baseline CRP and COPD | 335 | Early progression | Intermediate vs Favorable recovery | Adjusted OR | 1.83 (0.82-4.07) | 0.138 |
| Complete-case adjustment for baseline CRP and COPD | 335 | Early progression | Persistent failure vs Favorable recovery | Adjusted OR | 2.48 (1.21-5.07) | 0.013 |
| Complete-case adjustment for baseline CRP and COPD | 335 | Progression-free survival | Intermediate vs Favorable recovery | Adjusted HR | 2.20 (1.48-3.28) | <0.001 |
| Complete-case adjustment for baseline CRP and COPD | 335 | Progression-free survival | Persistent failure vs Favorable recovery | Adjusted HR | 3.15 (2.19-4.52) | <0.001 |
| Complete-case adjustment for baseline CRP and COPD | 335 | Overall survival | Intermediate vs Favorable recovery | Adjusted HR | 1.39 (0.92-2.09) | 0.114 |
| Complete-case adjustment for baseline CRP and COPD | 335 | Overall survival | Persistent failure vs Favorable recovery | Adjusted HR | 1.11 (0.76-1.62) | 0.595 |

Favorable recovery was the reference group. Baseline CRP adjustment used complete cases with available baseline CRP.

**Supplementary Table S7. Panel B. Treatment-subset analyses**

| **Analysis** | **n used** | **Endpoint** | **Comparison** | **Measure** | **Estimate** | **P value** |
| --- | --- | --- | --- | --- | --- | --- |
| ICI + chemotherapy only | 295 | Durable clinical benefit | Intermediate vs Favorable recovery | Adjusted OR | 0.50 (0.27-0.93) | 0.029 |
| ICI + chemotherapy only | 295 | Durable clinical benefit | Persistent failure vs Favorable recovery | Adjusted OR | 0.35 (0.20-0.63) | <0.001 |
| ICI + chemotherapy only | 295 | Early progression | Intermediate vs Favorable recovery | Adjusted OR | 1.41 (0.61-3.27) | 0.424 |
| ICI + chemotherapy only | 295 | Early progression | Persistent failure vs Favorable recovery | Adjusted OR | 1.85 (0.86-3.96) | 0.113 |
| ICI + chemotherapy only | 295 | Progression-free survival | Intermediate vs Favorable recovery | Adjusted HR | 2.34 (1.52-3.60) | <0.001 |
| ICI + chemotherapy only | 295 | Progression-free survival | Persistent failure vs Favorable recovery | Adjusted HR | 3.07 (2.07-4.56) | <0.001 |
| ICI + chemotherapy only | 295 | Overall survival | Intermediate vs Favorable recovery | Adjusted HR | 1.36 (0.89-2.09) | 0.155 |
| ICI + chemotherapy only | 295 | Overall survival | Persistent failure vs Favorable recovery | Adjusted HR | 1.10 (0.74-1.64) | 0.634 |
| No antiangiogenic therapy | 351 | Durable clinical benefit | Intermediate vs Favorable recovery | Adjusted OR | 0.51 (0.29-0.89) | 0.018 |
| No antiangiogenic therapy | 351 | Durable clinical benefit | Persistent failure vs Favorable recovery | Adjusted OR | 0.33 (0.20-0.57) | <0.001 |
| No antiangiogenic therapy | 351 | Early progression | Intermediate vs Favorable recovery | Adjusted OR | 1.60 (0.76-3.38) | 0.218 |
| No antiangiogenic therapy | 351 | Early progression | Persistent failure vs Favorable recovery | Adjusted OR | 1.87 (0.94-3.72) | 0.075 |
| No antiangiogenic therapy | 351 | Progression-free survival | Intermediate vs Favorable recovery | Adjusted HR | 2.35 (1.60-3.46) | <0.001 |
| No antiangiogenic therapy | 351 | Progression-free survival | Persistent failure vs Favorable recovery | Adjusted HR | 3.27 (2.29-4.69) | <0.001 |
| No antiangiogenic therapy | 351 | Overall survival | Intermediate vs Favorable recovery | Adjusted HR | 1.36 (0.92-2.00) | 0.121 |
| No antiangiogenic therapy | 351 | Overall survival | Persistent failure vs Favorable recovery | Adjusted HR | 1.19 (0.82-1.72) | 0.350 |
| Antiangiogenic-containing therapy | 58 | Durable clinical benefit | Intermediate vs Favorable recovery | Adjusted OR | 0.58 (0.10-3.35) | 0.545 |
| Antiangiogenic-containing therapy | 58 | Durable clinical benefit | Persistent failure vs Favorable recovery | Adjusted OR | 0.28 (0.05-1.72) | 0.170 |
| Antiangiogenic-containing therapy | 58 | Early progression | Intermediate vs Favorable recovery | Adjusted OR | 0.64 (0.06-7.04) | 0.712 |
| Antiangiogenic-containing therapy | 58 | Early progression | Persistent failure vs Favorable recovery | Adjusted OR | 2.86 (0.48-17.00) | 0.248 |
| Antiangiogenic-containing therapy | 58 | Progression-free survival | Intermediate vs Favorable recovery | Adjusted HR | 3.04 (0.90-10.25) | 0.073 |
| Antiangiogenic-containing therapy | 58 | Progression-free survival | Persistent failure vs Favorable recovery | Adjusted HR | 8.37 (2.35-29.81) | 0.001 |
| Antiangiogenic-containing therapy | 58 | Overall survival | Intermediate vs Favorable recovery | Adjusted HR | 2.05 (0.53-7.93) | 0.296 |
| Antiangiogenic-containing therapy | 58 | Overall survival | Persistent failure vs Favorable recovery | Adjusted HR | 0.85 (0.22-3.37) | 0.821 |

Treatment-subset analyses should be interpreted cautiously because subgroup sample sizes were limited, particularly for antiangiogenic-containing therapy.

**Supplementary Table S8. Effect-modification tests by PD-L1 status, treatment pattern, and antiangiogenic exposure**

| **Endpoint** | **Modifier** | **Interaction P value** |
| --- | --- | --- |
| Durable clinical benefit | PD-L1 TPS category | 0.436 |
| Early progression | PD-L1 TPS category | 0.728 |
| Progression-free survival | PD-L1 TPS category | 0.679 |
| Overall survival | PD-L1 TPS category | 0.826 |
| Durable clinical benefit | First-line treatment pattern | 0.993 |
| Early progression | First-line treatment pattern | 0.877 |
| Progression-free survival | First-line treatment pattern | 0.84 |
| Overall survival | First-line treatment pattern | 0.425 |
| Durable clinical benefit | Antiangiogenic exposure | 0.915 |
| Early progression | Antiangiogenic exposure | 0.368 |
| Progression-free survival | Antiangiogenic exposure | 0.688 |
| Overall survival | Antiangiogenic exposure | 0.755 |

Interaction P values were obtained from models including a dynamic signature by modifier interaction term. These tests evaluate statistical evidence of effect modification and are not powered to prove absence of heterogeneity.

# Supplementary Figures

Supplementary Figure S1. Missingness profile for variables with at least one missing value.

Supplementary Figure S2. Longitudinal NLR, PNI, and ALI trajectories from baseline to cycle 2 and cycle 4 according to the rule-derived dynamic recovery signature. Lines show medians and ribbons show IQRs. Cycle 4 values are available-case summaries.

Supplementary Figure S3. Adjusted associations of individual dynamic components with durable benefit, early progression, progression-free survival, and overall survival.

Supplementary Figure S4. Safety endpoints according to the rule-derived dynamic recovery signature, including any immune-related adverse event, treatment discontinuation, grade 3 or higher adverse event, and immune-related pneumonitis.

# Abbreviations

AE, adverse event; ALI, advanced lung cancer inflammation index; AUC, area under the receiver operating characteristic curve; CRP, C-reactive protein; DCB, durable clinical benefit; EP, early progression; HR, hazard ratio; ICI, immune checkpoint inhibitor; IQR, interquartile range; NLR, neutrophil-to-lymphocyte ratio; NSCLC, non-small cell lung cancer; OR, odds ratio; OS, overall survival; PD-L1, programmed death-ligand 1; PFS, progression-free survival; PNI, prognostic nutritional index; ROC, receiver operating characteristic; TPS, tumor proportion score.
